# Epidemiology of Healthcare-Associated Infections Caused by Multidrug-Resistant Bacteria and Antimicrobial Resistance Patterns in a Romanian Tertiary Care Hospital

**DOI:** 10.3390/jcm15020667

**Published:** 2026-01-14

**Authors:** Andreea Mihaela Sandu, Corneliu Ovidiu Vrancianu, Ana-Catalina Tantu, Vasilica Mihaela Dumitrache, Daniel Diaconescu, Roxana-Elena Cristian, Andreea Marcu, Monica Marilena Tantu

**Affiliations:** 1 Doctoral School, Carol Davila University of Medicine and Pharmacy, Eroii Sanitari 8, District 5, 050474 Bucharest, Romania; andreea-mihaela.sandu@drd.umfcd.ro; 2 The County Emergency Hospital, Aleea Spitalului 36, 110283 Pitesti, Romania; 3 The Research Institute of the University of Bucharest (ICUB), Șoseaua Panduri 90, District 5, 050663 Bucharest, Romania; roxana.cristian@incdsb.ro; 4 National Institute of Research and Development for Biological Sciences, 296 Splaiul Independentei, District 6, 060031 Bucharest, Romania; 5 Doctoral School, University of Medicine and Pharmacy of Craiova, Petru Rareș 2, 200349 Craiova, Romania; ana.tantu@umfcv.ro; 6 Emergency Clinical County Hospital of Bucharest, Calea Floreasca 8, 014461 Bucharest, Romania; 7 Department of Mathematics and Informatics, Faculty of Sciences, Physical Education and Informatics, Pitesti University Center, National University of Science and Technology Politehnica Bucharest, 060042 Bucharest, Romania; mihaela.dumitrache@upb.ro; 8 Department of Medical Assistance and Physical Therapy, Pitesti University Center, Târgu din Vale 1, 110040 Pitesti, Romania; daniel.diaconescu@upb.ro (D.D.); marilena.tantu@upb.ro (M.M.T.); 9 Faculty of Science, Physical Education and Informatics, National University of Science and Technology, Politehnica, Splaiul Independenței 313, District 6, 060042 Bucharest, Romania; 10 Faculty of Medicine, “Carol Davila” University of Medicine and Pharmacy, 020021 Bucharest, Romania; andreea.marcu@drd.umfcd.ro

**Keywords:** hospital-acquired infections, hospital infection surveillance, antimicrobial stewardship, multidrug-resistant bacteria

## Abstract

**Background/Objectives:** Healthcare-associated infections (HAIs), particularly those caused by multidrug-resistant (MDR) bacteria, remain a major challenge for Romanian hospitals. This study aimed to evaluate the epidemiological burden of MDR-related HAIs and to characterize the distribution of MDR bacterial isolates and their antimicrobial resistance patterns over four consecutive semesters in a Romanian tertiary care hospital. **Methods:** A retrospective study was conducted using data from the Electronic Registry of HAIs, clinical observation sheets, and microbiology laboratory records. An epidemiological analysis was performed on patients diagnosed with MDR-related HAIs, while a separate microbiological analysis included all MDR bacterial isolates identified during the study period. Descriptive and comparative statistical analyses were applied to assess temporal trends, pathogen distribution, and resistance profiles. **Results:** Of the 327 HAIs identified, 56 cases (17.13%) were caused by MDR bacteria. Most MDR-HAIs originated from the Intensive Care Unit (≈60%), with *Acinetobacter baumannii* and *Klebsiella* spp. as the predominant pathogens. Overall mortality among patients with MDR-HAIs was high (51.79%), particularly in infections caused by *A. baumannii* and *K. pneumoniae*. Microbiological analysis of MDR isolates (*n* = 406) revealed consistently high resistance rates to ciprofloxacin, cefepime, and ceftazidime, exceeding 95% in 2023–2024, while resistance to carbapenems surpassed 90% by the end of the study period. Temporal variability in MDR burden was observed across semesters, suggesting an influence of clinical and institutional factors. **Conclusions:** MDR-related HAIs represent a significant and persistent problem in Romanian acute-care hospitals, particularly in Intensive Care Units. The dominance of carbapenem-resistant *A. baumannii* and extended-spectrum beta-lactamase-producing and carbapenem-resistant *Klebsiella* spp. highlights the urgent need for strengthened antimicrobial stewardship, enhanced microbiological surveillance, and reinforced infection prevention strategies.

## 1. Introduction

Healthcare-associated infections (HAIs) are defined as infections that were neither present nor incubating at the time of hospital admission and typically become evident 48 h or more after admission or occur in association with medical procedures or care received in healthcare settings. HAIs have significant consequences for both patients and healthcare facilities, representing an important cause of morbidity and mortality worldwide [1].

HAIs generate additional suffering for patients, prolonging hospitalization and increasing the need for care [2], requiring much greater human and material resources than would have been necessary in the case of an uncomplicated evolution [3]. They affect health systems and, implicitly, healthcare facilities by increasing the costs associated with the care of patients who develop HAIs [4], but also by influencing some performance indicators of hospital management (average length of stay, bed utilization rate) [5], with an emphasis on those of quality and patient safety (adverse events, complaints, mortality rate) [6]. The most common HAIs are catheter-associated urinary tract infections (CAUTI), surgical site infections (SSI), central line-associated bloodstream infections (CLABSI), ventilator-associated pneumonia, and *Clostridioides difficile* infections (CDI) [7]. Various studies show that the most common microorganisms involved in such cases are methicillin-resistant *Staphylococcus aureus* (MRSA), *Pseudomonas aeruginosa*, *Klebsiella pneumoniae*, *Escherichia coli*, and *Acinetobacter baumannii* [8,9,10].

In Romania, HAIs represent a much-underestimated pathology, with officially reported prevalence rates of only 0.2–0.25% despite evidence suggesting a much higher real burden due to significant underreporting [11]. More accurate estimates indicate a prevalence of 2.6%, corresponding to approximately 100,000 cases annually [11]. The most problematic HAIs in Romanian hospitals are CDI and carbapenem-resistant *Enterobacterales* (CRE) infections, which have been increasing steadily and are associated with high healthcare costs [12]. Several national and regional investigations also highlight elevated resistance profiles among isolates from respiratory, urinary, bloodstream, and surgical infections, with some of the highest proportions of carbapenemase-producing *Enterobacterales*, *A. baumannii*, *P. aeruginosa*, *S. aureus*, and *K. pneumoniae* [13,14,15,16,17,18,19,20,21,22]. Although national surveillance has improved following the implementation of standardized reporting procedures, the officially reported HAI incidence remains below European averages, while retrospective and epidemiological studies consistently demonstrate a substantially higher true prevalence in Romanian healthcare settings [23].

There is a mutual influence and a relationship of determination between HAIs and antibiotic resistance [24]. Very often, an HAI is caused by a multidrug-resistant (MDR) bacterium. This factor can be challenging to address in settings where new antibiotics are not available [10]. In general, it is considered that antibiotic resistance arises against the background of injudicious and inappropriate antibiotic use in the general population and the community [6]. However, it should also be noted that antibiotics are used to treat nosocomial infections, usually in combination, intensively, and for prolonged periods, thereby selecting for antibiotic-resistant microorganisms [24].

HAIs are not eliminated, even in the most developed and well-funded health systems. Although they are associated with significant costs, the only option to combat these infections is prevention and control, namely the implementation and development of efficient prevention, surveillance, and control management at the level of each health unit [25].

For the prevention of HAIs, an important aspect is knowledge of the types of microorganisms present in the hospital environment (surfaces, objects, and aeromicroflora), as well as the microbial load patients have at the time of hospital admission [26]. Thus, the importance of infectious screening upon admission and during transfers, especially to/from the intensive care unit (ICU), is highlighted by taking skin samples, nasopharyngeal exudate, and wound cultures, especially if there are clear signs of infection [27]. In addition, the assessment of infectious risk is complemented by using the Carmeli score, which helps differentiate between community-acquired and HAIs and supports the selection of appropriate empirical antibiotic therapy [28].

Identifying pathogens or germs in infectious outbreaks, pre-existing admissions, and knowledge of antibiotic resistance characteristics, including sensitivity to various groups of antibiotics, respectively, indicates the directions of action in the preventive management of HAIs [26]. Preventive management could include proper cohorting of patients, rigorous adherence to standard precautions, and the application of specific measures targeted to the transmission route, including antibiotic stewardship and the formulation of protocols for antibiotic prophylaxis and therapy [25].

This study was conducted in a large tertiary-care emergency hospital that serves as a primary referral center for acute and critically ill patients in Argeș County and neighboring regions. The hospital manages a high volume of patients requiring urgent and prolonged hospitalization, including intensive care and high-complexity medical and surgical services, settings known to represent key reservoirs for HAIs and antimicrobial resistance, particularly in countries with limited longitudinal data.

The novelty of this analysis lies in its longitudinal evaluation of MDR-related HAIs across successive semesters, allowing assessment of temporal shifts in pathogen distribution, ward-specific burden, and resistance patterns in a real-world emergency care context. Unlike previous Romanian reports, which are predominantly cross-sectional or pathogen-specific, this study integrates microbiological, clinical, and epidemiological data over time, providing a dynamic perspective on MDR-HAIs in a tertiary hospital setting and contributing locally relevant evidence to support infection control and antimicrobial stewardship efforts.

The present study aims to characterize the impact of MDR bacteria in a Romanian emergency hospital by integrating epidemiological and microbiological data. We analyzed the temporal distribution of HAIs caused by MDR pathogens between 1 July 2022 and 30 June 2024, stratified by semester. In parallel, we examined the distribution of MDR bacterial isolates identified through routine bacteriological testing and described their antimicrobial resistance profiles during the same study period. These findings provide a basis for targeted infection prevention measures and support the optimization of antimicrobial stewardship strategies within the hospital setting.

## 2. Materials and Methods

A retrospective analysis was conducted using data from the Electronic Registry of HAIs, clinical observation sheets, and laboratory records. Data were extracted from the hospital’s electronic system and statistically analyzed to determine correlations between infection rates and resistance profiles.

### 2.1. Ethical Considerations and Study Design

This study was conducted in compliance with ethical standards and was approved by the Ethics Subcommittee for Scientific Research of the Carol Davila University of Medicine and Pharmacy, under protocol number 30316/24 October 2025.

This was a retrospective, observational, single-center study conducted using routinely collected clinical, epidemiological, and microbiological data. The study aimed to describe temporal trends in MDR-related HAIs, pathogen distribution, and antimicrobial resistance patterns, without intervention or modification of standard clinical practice.

### 2.2. Data Sources and Institutional Framework

Data were obtained from multiple institutional sources, including the electronic HAIs registry, general clinical observation records (GCORs), individual case records, the hospital information system, and the microbiology laboratory database. These sources were used to retrieve epidemiological, clinical, and microbiological data relevant to the study objectives. All data were anonymized prior to analysis and organized in a structured format that allowed for integrated epidemiological and microbiological evaluation.

### 2.3. HAIs Surveillance

HAIs were identified in accordance with institutional surveillance protocols and national regulations in force during the study period. Case identification was based on routinely collected clinical and epidemiological data recorded by the hospital infection prevention and control service. Only confirmed cases were included in the epidemiological analysis.

Among all patients diagnosed with HAIs during the study period, cases caused by MDR bacterial pathogens were identified based on microbiological confirmation and antimicrobial susceptibility testing. These cases were included in the temporal epidemiological analysis stratified by semester.

### 2.4. Inclusion and Exclusion Criteria

#### 2.4.1. Clinical Inclusion and Exclusion Criteria for MDR-Related HAIs

Patients were eligible for inclusion in the epidemiological analysis if they were diagnosed with HAIs during the study period (1 July 2022 and 30 June 2024), in accordance with institutional surveillance protocols and national definitions. Case identification was based on routinely collected clinical and epidemiological data.

Among these cases, infections caused by MDR bacterial pathogens were confirmed microbiologically and tested for antimicrobial susceptibility. These cases constituted the subgroup used for the analysis of MDR-related HAIs.

Patients with evidence of bacterial colonization or asymptomatic carriage, as well as cases in which no epidemiological link could be established between the infection and the current hospitalization episode, were excluded from the epidemiological analysis.

#### 2.4.2. Microbiological Inclusion and Exclusion Criteria for MDR Isolates

For the microbiological analysis, bacteriological samples processed by the hospital microbiology laboratory between 1 July 2022 and 30 June 2024 were eligible for inclusion. Only bacterial isolates for which complete antimicrobial susceptibility testing results were available were considered.

Antimicrobial susceptibility testing was required to be performed in accordance with current laboratory standards and applicable EUCAST guidelines. Isolates were included in the final microbiological analysis only if they fulfilled the criteria for multidrug resistance, defined as non-susceptibility to at least one antimicrobial agent in three or more antimicrobial classes.

Bacteriological samples interpreted as contaminated were excluded from the analysis. Isolates without antimicrobial susceptibility testing, isolates that did not meet the predefined resistance criteria, and cases with incomplete data or insufficient linkage between the biological sample and the corresponding susceptibility results were also excluded.

The epidemiological analysis focused on patients with HAIs caused by MDR pathogens, whereas the microbiological analysis included all MDR bacterial isolates identified during the study period.

### 2.5. The Electronic Registry of HAIs

The electronic HAIs registry is integrated into the Hippocrates IT system, allowing for centralized surveillance and monitoring of all cases reported within the healthcare facility. Access is secured, allowing only authorized users. The registry contains the following essential information: the department of origin of the case, the number of the GCOR, the patient’s age and sex, the date of admission and the date of infection detection, the detection method (active or passive), the isolated germ, the microbial resistance profile (including MDR strains), the type of infection or the location of colonization, the origin of the infection, and the final classification. The registry enables the extraction and analysis of cases reported within a specific period and is actively utilized by the Healthcare-Associated Infection Prevention Service (SPIAAM).

Data extracted from the electronic HAIs registry were used exclusively for epidemiological analysis of healthcare-associated infections, while detailed microbiological analyses were performed using data directly from microbiology laboratory records.

### 2.6. Clinical Observation Sheets of Patients Diagnosed with HAIs

GCORs are the primary documents used to record medical information for each patient. They include identification data, demographic information, history, symptoms, clinical and laboratory investigations, treatments administered, patient progress during hospitalization, and complications. In the case of patients with HAI, GCORs provide relevant information regarding the time of infection onset, the application of invasive devices or surgical interventions, antimicrobial treatment history, the presence of associated risk factors, and discharge status. These records are essential for validating reported cases and correlating clinical information with microbiological and epidemiological information.

Data extracted from GCORs were used to support the clinical and epidemiological characterization of HAIs, while microbiological analyses were based on laboratory records and antimicrobial susceptibility testing data.

### 2.7. Laboratory Records Detailing Microbial Isolates and Antibiotic Susceptibility Profiles

Microbiological data were extracted from both the medical laboratory computer system and physical records, with the support of medical informatics specialists. During the study period, a total of 41,457 bacteriological samples were processed in the hospital microbiology laboratory. Of these, 4374 samples yielded positive cultures. Antimicrobial susceptibility testing was performed for 1550 isolates that showed resistance to at least one antimicrobial agent. Based on predefined inclusion criteria, 406 bacterial isolates were classified as MDR and were included in the microbiological analysis.

The information obtained included the identification of the pathogens, the identification of bacterial pathogens isolated from clinical samples, the etiological confirmation of the infection, and the antimicrobial susceptibility profiles of each isolate. HAI cases were included only when microbiological findings were concordant with clinical evidence of infection. This included compatible clinical signs and symptoms, laboratory or inflammatory markers, radiological findings when applicable, and the initiation of targeted antimicrobial therapy by the treating physician. Isolates interpreted as colonization or asymptomatic carriage, particularly for organisms known to colonize skin or mucosal surfaces, were excluded from the analysis. ICD-10 codes were used for case identification and verification but were not applied as the sole diagnostic criterion.

Antibiotic resistance was analyzed, with the identification of MDR strains where applicable. These data were essential for the microbiological characterization of MDR isolates and, where applicable, for the epidemiological analysis of HAIs.

Antimicrobial susceptibility testing (AST) was performed in the hospital microbiology laboratory using routine diagnostic methods. Bacterial identification and AST were conducted according to the European Committee on Antimicrobial Susceptibility Testing (EUCAST) guidelines applicable at the time of testing. Susceptibility results were interpreted using EUCAST clinical breakpoints. Internal quality control procedures were routinely applied in accordance with laboratory standard operating protocols. Resistance rates were calculated based on the number of tested isolates for each antimicrobial agent, as routinely reported by the laboratory. Denominators therefore vary depending on antimicrobial availability and testing panels.

MDR organisms were defined according to the international consensus criteria proposed by Magiorakos et al. [29] as isolates exhibiting non-susceptibility to at least one agent in three or more antimicrobial classes. Resistance classification was applied at the isolate level, based on AST results obtained for each clinical isolate. Although some isolates exhibited resistance to a large number of individual antibiotics, the absolute number of resistant agents was not used as a formal criterion for MDR classification, as this parameter depends on the AST panels and does not represent a standardized resistance category. Extensively drug-resistant (XDR) and pandrug-resistant (PDR) classifications were not systematically assessed and are therefore not used for categorical interpretation in this study.

All bacterial isolates meeting the criteria for multidrug resistance during the study period were included in the microbiological analysis, regardless of infection classification or hospital ward. Each patient was included once, based on the clinically relevant MDR isolate.

### 2.8. Data Processing and Statistical Analysis

Data were analyzed primarily using descriptive statistics. Categorical variables are reported as counts and percentages, and continuous variables as mean ± SD or median (IQR), as appropriate. Normality was assessed using the Jarque–Bera test. For clinically meaningful predefined comparisons, continuous variables were compared using the independent-samples *t*-test when normally distributed or the Mann–Whitney U test otherwise. Categorical variables were compared using the chi-square test or Fisher’s exact test when expected cell counts were small. Statistical testing was avoided for exploratory subgroup analyses with very small sample sizes (e.g., *n* ≤ 5 per category), and such results are presented descriptively. A two-sided *p*-value < 0.05 was considered statistically significant; very small *p*-values are reported as *p* < 0.001. No formal correction for multiple comparisons was applied because several analyses were descriptive and exploratory; therefore, inferential findings were interpreted cautiously. Analyses were performed using SPSS (version 27.0.0) and Microsoft Excel (version 2021).

## 3. Results

### 3.1. Epidemiological Trends of MDR-Related HAIs

#### 3.1.1. Patient Characteristics

During the study period, a total of 327 HAI cases were identified, of which 56 cases (17.13%) were caused by MDR bacteria (Table 1).

An increasing trend in the proportion of MDR-related HAIs was observed over time, with no MDR cases recorded in July–December 2022, followed by an increase to 14% in January–June 2023 and a peak of 36.2% (25/69 cases) in July–December 2023 (Table 1). The proportion subsequently decreased to 24% (18/75) in January–June 2024.

In the study cohort, female patients accounted for a higher proportion of HAI cases (57.1%, *n* = 32) compared with male patients (42.9%, *n* = 24). This sex-related difference was statistically significant (*p* < 0.001).

**Figure 1:**
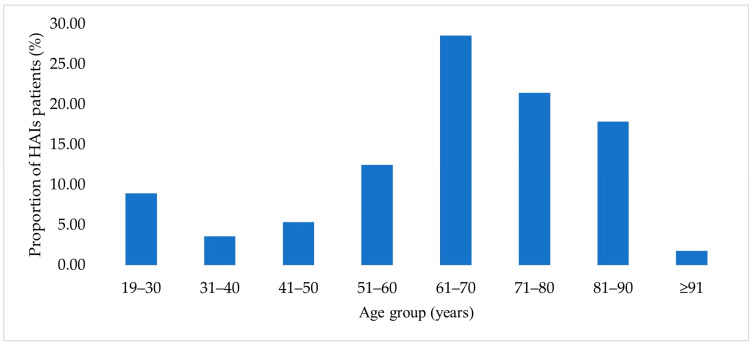
Age distribution of patients with HAIs.

As shown in Figure 1, the highest proportion of patients was in the 61–70-year age group (16 patients, 28.6%), which was significantly higher than in the 71–80-year group (12 patients, 21.4%; *p* = 0.002).

The mean age of the 56 patients included in the analysis was 63.9 ± 18.6 years, with a median age of 68 years and an age range of 21–95 years (Table 2). Women had a significantly higher mean age compared to men (69.1 vs. 57 years, *p* = 0.011, *t*-test).

Patients from urban areas had a higher mean age compared to those from rural areas (66.7 vs. 60.6 years). Of the 56 patients included in the study, 30 (53.6%) were from urban areas, while 26 (46.4%) resided in rural areas (Table 3).

Most patients were hospitalized in the ICU (59%), followed by the surgical (23.2%) and medical wards (17.9%). The median age was lowest among ICU patients (60.0 years) and highest among those admitted to medical wards (71.0 years).

The ICU ward accounted for the highest proportion of cases (58.93%, 33 patients). The median age of patients admitted to the medical wards was higher than that observed in the ICU and surgical wards; however, no statistically significant differences were identified between ward-specific age distributions (Table 3). Given the distinct distribution of cases across hospital wards, ICU cases were analyzed and reported separately.

#### 3.1.2. Distribution of Isolated Pathogens in HAI Cases

*A. baumannii* (28.57%) and *K. pneumoniae* (26.79%) were the most frequently isolated pathogens during the study period. Together, these species accounted for the highest proportion of isolates compared with other bacterial pathogens identified in the cohort.

The distribution of MDR-HAI cases by bacterial strain and hospital ward (ICU, surgical, and medical) showed a predominance of ICU cases, accounting for 33 of 56 cases (58.9%) (Table 4). The most frequently isolated organisms were *A. baumannii* (7 cases) and *Acinetobacter* spp. (9 cases), followed by species belonging to the *Klebsiella genus*, including *K. ozaenae* and *K. pneumoniae.*

#### 3.1.3. Distribution of Comorbidities Among HAI Patients

**Figure 2:**
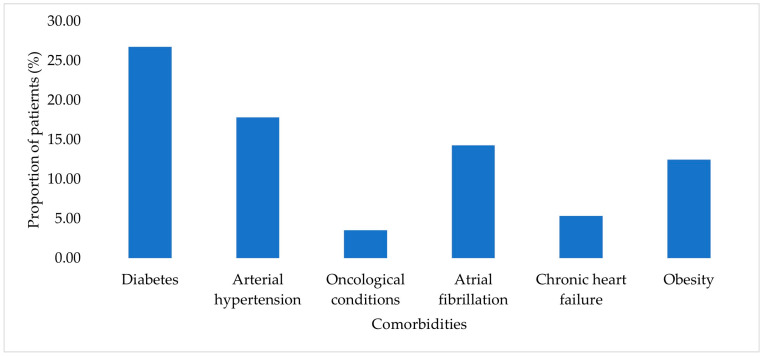
Prevalence of comorbidities among patients with HAIs.

Diabetes mellitus, arterial hypertension, atrial fibrillation, and obesity were among the most frequently reported comorbid conditions in the study cohort, with diabetes mellitus identified in 26.79% of cases. The distribution of comorbidities within the study cohort is presented in Figure 2. Each comorbidity was recorded individually for each patient.

#### 3.1.4. Structure of the Study Cohort According to Identified Pathogens and Presence of Comorbidities

The distribution of MDR pathogens according to the presence of major patient comorbidities is presented descriptively in Supplementary Table S1. The assessed comorbidities included arterial hypertension, chronic heart failure, obesity, diabetes mellitus, atrial fibrillation, and oncological conditions.

#### 3.1.5. Mortality Patterns and Pathogen Association in MDR-HAIs

Among the 56 patients with HAIs caused by MDR pathogens, 29 patients (51.79%) died during hospitalization. Most fatal outcomes occurred in patients admitted to the ICU.

**Figure 3:**
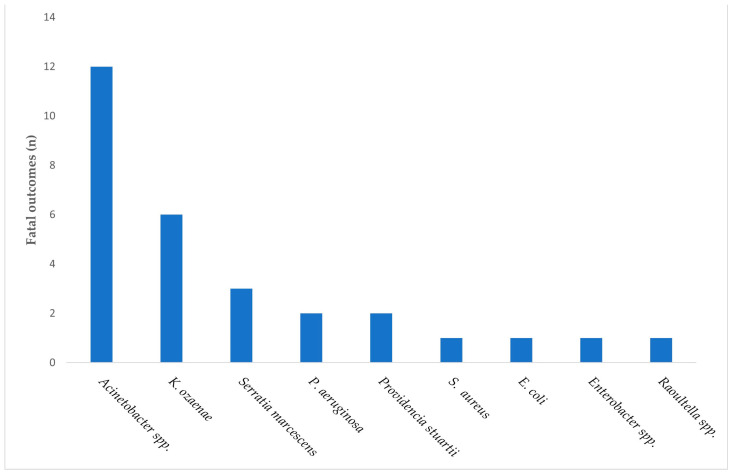
Distribution of fatal outcomes among patients with MDR-associated HAIs, stratified by the causative pathogen.

When mortality was descriptively analyzed according to the causative pathogen, the highest number of fatal outcomes was observed among cases associated with *Acinetobacter* spp., followed by *K. ozaenae*, *S. aureus*, and *P. aeruginosa* (Figure 3).

In addition to the epidemiological analysis of MDR-HAIs, a separate microbiological analysis was conducted focusing exclusively on bacterial isolates classified as MDR during the study period.

## 4. Characterization of MDR Bacterial Isolates and Associated Patient Characteristics

### 4.1. Patient Characteristics

**Figure 4:**
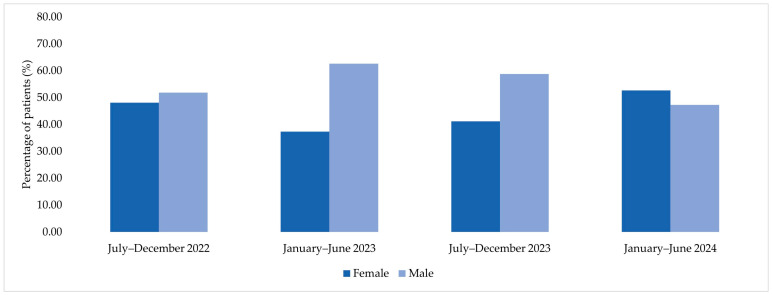
Sex distribution of patients corresponding to MDR bacterial isolates across the four analyzed semesters (2022–2024).

Among patients corresponding to MDR bacterial isolates included in the microbiological analysis, the distribution by sex varied across the four analyzed semesters. In the second semester of 2022, among the 27 patients included, 48.15% were female and 51.85% were male. In the first semester of 2023, among 83 patients, males accounted for 62.65% of cases, while females represented 37.35%. A similar distribution was observed in the second semester of 2023, when 58.78% of the 131 patients were male and 41.22% were female. In the first semester of 2024, among 165 patients, 52.73% were female and 47.27% were male (Figure 4).

**Figure 5:**
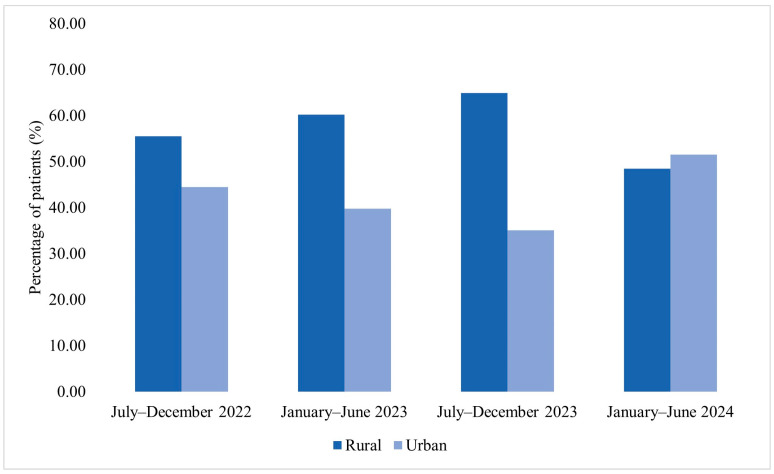
Distribution of patients corresponding to MDR bacterial isolates by area of residence across the four analyzed semesters (2022–2024).

Among patients corresponding to MDR bacterial isolates included in the microbiological analysis, the distribution by area of residence varied across the four analyzed semesters (Figure 5). In July–December 2022, among the 27 patients included, 55.6% were from rural areas and 44.4% from urban areas. In January–June 2023, rural residents accounted for 60.2% of cases, while 39.8% were from urban settings. A similar distribution was observed in July–December 2023, when 64.9% of patients originated from rural areas and 35.1% from urban areas. In January–June 2024, 48.4% of patients were from rural areas and 51.5% from urban areas.

Analysis of age distribution among patients corresponding to MDR bacterial isolates over the study period (2022–2024) revealed a progressive increase in mean age. In July–December 2022, the mean age was 58 ± 20.1 years, increasing to 67 ± 20 years in January–June 2023. This was followed by a mean age of 64 ± 15.5 years in July–December 2023, reaching 69.2 ± 11.9 years in January–June 2024 (Table 5). Across all semesters, median values were close to the corresponding means, indicating a relatively symmetrical age distribution within each period.

**Figure 6:**
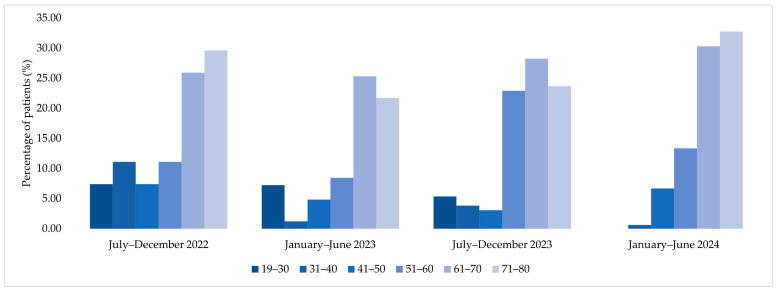
Age group distribution of patients corresponding to MDR bacterial isolates across the four analyzed semesters (2022–2024).

Age group analysis among patients corresponding to MDR bacterial isolates showed a predominance of older age categories, particularly patients aged 61–70 and 71–80 years, during 2022 and 2023. In contrast, January–June 2024 displayed a more evenly distributed age profile across adult age groups (Figure 6).

Across all analyzed semesters, patients from urban areas consistently exhibited higher median ages compared with those from rural areas (Table 6). This pattern was observed in each semester and appeared to be independent of sex distribution.

In contrast, differences in age between female and male patients were modest and varied across time, without a consistent trend (Table 7). Overall, area of residence showed a more stable association with patient age than sex, with urban patients tending to be older than rural patients throughout the study period.

**Figure 7:**
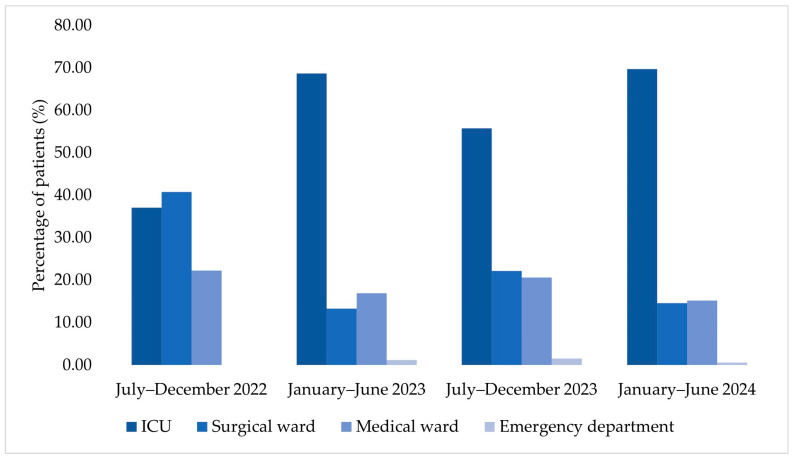
Temporal distribution of hospitalized patients according to department type.

The ICU accounted for an increasing number of cases among patients corresponding to MDR bacterial isolates across the four analyzed semesters (Figure 7). In July–December 2022, the proportion of samples from the surgical ward slightly exceeded that from the ICU. Beginning with January–June 2023, the ICU accounted for the largest proportion of cases compared with both medical and surgical wards, a pattern that persisted through July–December 2023 and January–June 2024.

Alongside this epidemiological shift, an increase in the average age of ICU patients was observed over the study period, rising from 53.1 years in July–December 2022 to 68.85 years in January–June 2024.

Changes in gender distribution were also observed over time, with an increase in the number of female patients admitted to the ICU across successive semesters. Patients from rural areas accounted for a substantial proportion of ICU admissions in each analyzed semester.

### 4.2. Bacterial Distribution and Antibiotic Resistance Trends Across Study Semesters

In July–December 2022, the distribution of MDR bacterial isolates was relatively balanced across the ICU, surgical, and medical wards, with the highest proportion observed in the surgical ward (40.74%). *A. baumannii* and *Klebsiella* spp. were identified among the isolates collected during this period (Supplementary Figure S1).

In January–June 2023, an increase in the proportion of isolates originating from the ICU was observed, accounting for 68.67% of all identified pathogens. During this semester, *Acinetobacter* spp. and *Raoultella* spp. accounted for the highest number of isolates, alongside *Enterobacter* spp., *E. coli*, and *Klebsiella* spp. (Supplementary Figure S2).

In July–December 2023, the ICU remained the primary source of isolates, representing 55.73% of cases. A wider range of bacterial genera was identified during this period, including *Burkholderia cepacia* and *Yersinia* spp., while MDR Gram-negative bacteria accounted for the majority of isolates (Supplementary Figure S3).

**Figure 8:**
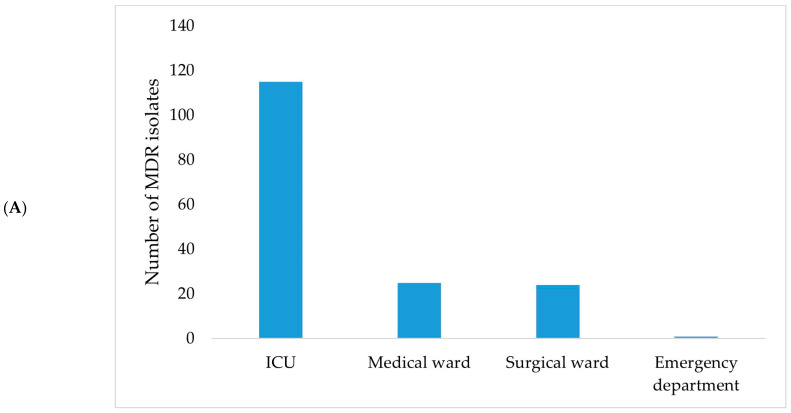

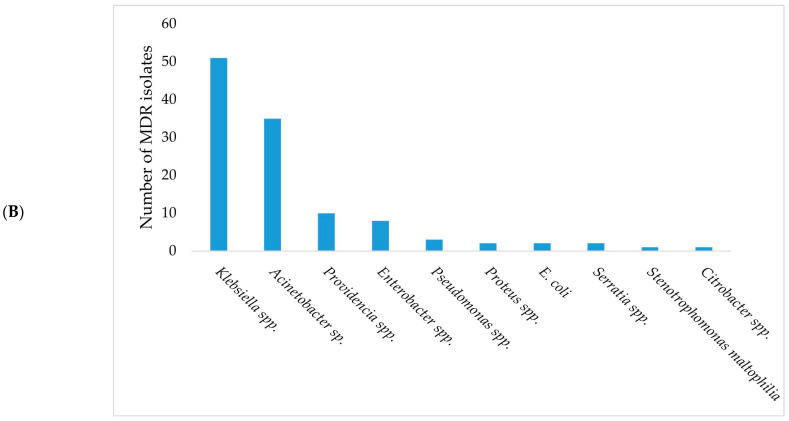
Distribution of MDR bacterial isolates by hospital ward (**A**) and bacterial genus (**B**) during January–June 2024.

In January–June 2024, the highest proportion of MDR isolates originated from the ICU (69.70%), with *Klebsiella* spp. (30.91%) and *Acinetobacter* spp. (21.21%) being the most frequently identified genera (Figure 8)*. Stenotrophomonas maltophilia* was also identified during this period.

#### 4.2.1. Antibiotic Resistance Patterns in Predominant Bacterial Strains

Across the 13 bacterial genera identified among MDR isolates included in the microbiological study group (*n* = 406), resistance to multiple antimicrobial classes was observed throughout the study period (2022–2024). The distribution of MDR isolates by bacterial genus, together with the key resistant antimicrobial classes and the presence of carbapenem resistance, is summarized in Table 8.

Overall, resistance to β-lactams, fluoroquinolones, and aminoglycosides was commonly observed across most genera, while carbapenem resistance was detected in several clinically relevant pathogens, particularly during the later semesters of the study period. The frequency of MDR isolates and the spectrum of resistant antimicrobial classes varied by bacterial genus and over time (Table 8). Temporal patterns are reported descriptively based on observed distribution across semesters and do not reflect formal statistical trend analyses.

#### 4.2.2. Antibiotic Resistance Profiles by Individual Antimicrobial Agent

The overall resistance profile observed among MDR bacterial isolates included in the microbiological study group across the four analyzed semesters showed high resistance rates to multiple antimicrobial agents. Resistance to ciprofloxacin, cefepime, and ceftazidime consistently exceeded 95% during 2023 and 2024. Resistance to carbapenems, including imipenem and meropenem, increased over time and exceeded 90% by the end of the study period (Supplementary Table S2).

Lower resistance rates (<10%) were observed for aztreonam, doxycycline, and fosfomycin trometamol across all analyzed semesters.

## 5. Discussion

This study examined the epidemiological characteristics of MDR-HAIs, together with the microbiological distribution and antimicrobial resistance patterns of MDR bacterial isolates, across four consecutive semesters in a Romanian tertiary-care hospital. By integrating clinical, demographic, and microbiological data, the analysis aimed to describe temporal trends, identify hospital wards with the highest MDR burden, and characterize the predominant MDR pathogens circulating in this setting.

HAIs and antimicrobial resistance remain major global public health challenges, particularly in acute-care and intensive care settings. Recent estimates indicate a substantial burden of HAIs and antibiotic-resistant infections across Europe and globally, with MDR Gram-negative organisms among the most critical threats in hospitalized patients [30,31]. Within this context, local longitudinal data are essential to inform infection prevention strategies and antimicrobial stewardship at the institutional level.

The findings of the present study are consistent with recent Romanian surveillance data, which indicate that MDR pathogens continue to pose a substantial challenge across healthcare settings. Similar to national reports, *A. baumannii* and *Klebsiella* spp. accounted for the largest proportion of MDR-HAIs in our cohort, with a pronounced predominance among ICU patients [19,20]. The high mortality observed, particularly in infections caused by *A. baumannii* and *K. pneumoniae*, mirrors patterns reported in Romanian cardiovascular and transplant centers, where MDR Gram-negative infections were major predictors of adverse outcomes [21]. The predominance of ICU-related cases further aligns with regional evidence highlighting the role of invasive procedures, prolonged device use, and extensive exposure to broad-spectrum antibiotics in sustaining MDR pathogen circulation [30].

A key finding of this study is the longitudinal shift in the distribution of MDR-related HAIs across hospital wards. While a relatively balanced distribution was observed in late 2022, a clear predominance of ICU-associated cases emerged from 2023 onward, with ICU-derived isolates accounting for nearly 70% of cases by early 2024. In parallel, an increase in the average age of ICU patients was observed over time, reflecting the growing representation of older and clinically complex patients in critical care settings [31]. The progressive increase in the average age of ICU patients observed in our cohort mirrors trends reported in large European and Asian ICU cohorts [32,33], which document a growing representation of older patients requiring critical care over time and consistently high mortality rates despite advances in ICU management.

Comorbidities were frequently observed among patients with MDR-related HAIs in our cohort, with arterial hypertension, heart failure, obesity, and diabetes mellitus being particularly prevalent. Similar associations between chronic comorbid conditions and increased susceptibility to HAIs have been reported in recent European single-center studies, where a higher comorbidity burden, often quantified using indices such as the Charlson score, was linked to prolonged hospitalization, ICU transfer, and adverse outcomes [34,35]. In line with our findings, diabetes mellitus has been consistently associated with impaired host defenses and increased infection risk, particularly in critically ill patients [36]. Moreover, ICU-based studies focusing on HAIs have identified diabetes, hypertension, and other chronic conditions as significant contributors to mortality, especially when combined with multidrug-resistant pathogens such as *K. pneumoniae* and *A. baumannii* [37]. Although oncological conditions were less frequent in our cohort, their clinical relevance is supported by previous reports highlighting the disproportionate risk of severe infections and poor outcomes in immunocompromised patients [35].

The mortality rate observed in this cohort was high, with fatal outcomes occurring predominantly among patients admitted to the ICU. Fatal cases were characterized by severe clinical profiles and intensive therapeutic exposure, features commonly reported in critically ill populations. *Acinetobacter* spp. accounted for a substantial proportion of fatal cases, consistent with recent hospital-based studies showing increased mortality associated with *A. baumannii* in ICU settings, particularly among patients with multiple comorbidities, prior broad-spectrum antibiotic exposure, and high rates of carbapenem resistance [37,38]. Similar patterns have also been reported in Romanian ICU cohorts, where *Acinetobacter* spp. predominated among older, mechanically ventilated patients [31,39]. Nevertheless, these findings derive from an unadjusted, descriptive analysis and should not be interpreted as evidence of independent, pathogen-specific mortality risk.

Demographic and temporal patterns provide additional contextual information regarding the MDR-HAI cohort. During 2022 and 2023, male patients and individuals from rural areas accounted for a higher proportion of cases, followed by a shift toward female predominance and a more balanced rural–urban distribution in 2024. Across all analyzed semesters, patients from rural areas exhibited a lower mean age compared with those from urban settings. Similar demographic patterns have been reported in Eastern European healthcare contexts and may reflect variability in referral pathways or access to specialized care [40,41,42].

From a microbiological perspective, the study reveals a dynamic pathogen landscape across the analyzed semesters. Earlier periods were characterized by greater species diversity, whereas later semesters showed increasing predominance of *Klebsiella* spp. and *Acinetobacter* spp. In addition, the sporadic identification of less frequently isolated pathogens, such as *B. cepacia*, *S. maltophilia*, and *Yersinia* spp., aligns with recent reports highlighting their emerging clinical relevance in critically ill and immunocompromised patients, often in association with extensive antimicrobial resistance and limited therapeutic options [43,44,45]. Although infrequent, their detection underscores the heterogeneity of MDR pathogens circulating in hospital environments and supports the need for sustained microbiological surveillance, particularly in ICU settings.

Gram-negative bacteria accounted for the majority of MDR-HAIs in the present cohort and therefore represent the main focus of the analysis, a pattern consistent with reports from Romanian and European tertiary-care hospitals where MDR Gram-negative pathogens predominate in ICU settings [31,46,47]. Nevertheless, Gram-positive organisms and fungi were also identified and merit brief consideration. *S. aureus* accounted for a smaller proportion of cases but was associated with severe outcomes in selected patients, in line with ICU-based studies reporting increased severity and mortality of *S. aureus*-associated infections, particularly among mechanically ventilated patients [46,47,48]. Fungal isolates, including *Candida* spp., were infrequently detected and were primarily observed in critically ill or immunocompromised patients, consistent with international ICU studies showing a lower incidence but high clinical relevance of *Candida*-associated infections in such populations [49,50]. Although the limited number of Gram-positive and fungal MDR cases precluded meaningful stratified analyses, their presence underscores the microbiological diversity of HAIs in tertiary-care hospital settings [51].

The resistance patterns observed in this study indicate a progressive expansion of multidrug resistance among several clinically significant Gram-negative pathogens over the 2022–2024 period. High resistance to β-lactams, fluoroquinolones, and aminoglycosides among *E. coli*, *Klebsiella* spp., and *Enterobacter* spp. is compatible with the circulation of ESBL-producing strains in the hospital setting. In addition, increasing resistance to carbapenems among *Acinetobacter* spp., *Klebsiella* spp., and *Pseudomonas* spp. was observed during the later semesters, suggesting sustained antimicrobial selective pressure, particularly in ICU-associated infections [20,31,42]. Less frequently isolated genera, including *Citrobacter* spp., *Providencia* spp., *Raoultella* spp., and *Serratia* spp., also exhibited broad resistance profiles involving multiple antimicrobial classes. Sporadic detection of intrinsically resistant organisms, such as *B. cepacia* and *S. maltophilia*, remains clinically relevant due to limited therapeutic options, especially in immunocompromised patients [41].

Importantly, the extensive resistance profiles observed in several isolates, including non-susceptibility to a broad range of tested antimicrobial agents, should be interpreted descriptively rather than as formal extensively drug-resistant (XDR) or pandrug-resistant (PDR) classifications. In this study, MDR was defined according to internationally accepted criteria, namely non-susceptibility to at least one agent in three or more antimicrobial classes, applied at the isolate level [29]. As the absolute number of resistant antibiotics depends on the AST panel used, this parameter was not used as a categorical definition but rather as an indicator of resistance breadth. These observations highlight the complexity of interpreting resistance profiles in clinical settings, particularly among critically ill patients.

At the level of individual antimicrobial agents, resistance to ciprofloxacin, cefepime, and ceftazidime exceeded 95% during 2023–2024, while resistance to carbapenems such as imipenem and meropenem surpassed 90% by the end of the study period. In contrast, aztreonam, doxycycline, and fosfomycin exhibited lower resistance rates across the analyzed semesters. Similar trends have been reported in recent Romanian surveillance studies, documenting increasing resistance to both first-line and last-resort antibiotics across multiple healthcare institutions [20,31,41]. The very high resistance rates observed for fluoroquinolones, cephalosporins, and carbapenems illustrate the therapeutic challenges associated with MDR pathogens in this setting. Although some agents, including aztreonam, doxycycline, and fosfomycin trometamol, exhibited lower resistance rates, their role in the management of severe MDR infections remains limited, particularly in critically ill patients.

Several limitations of this study should be acknowledged. First, mortality analyses were descriptive and unadjusted. Owing to the retrospective design and limited sample size, no multivariable or time-to-event analyses were performed, and outcomes were not adjusted for age, ICU admission, comorbidities, or disease severity. Accordingly, pathogen-specific mortality findings should be interpreted cautiously and cannot be considered independent or causal predictors. Second, the use of multiple subgroup comparisons within a relatively small cohort limits statistical inference; therefore, inferential testing was restricted to clinically meaningful comparisons, while other results are presented descriptively. Finally, as a single-center study, the findings may not be fully generalizable to other healthcare settings. Nevertheless, the longitudinal design and detailed microbiological characterization provide valuable insight into MDR-HAI dynamics in a tertiary-care hospital context.

The temporal fluctuations observed in the proportion of MDR-related HAIs may reflect the combined influence of infection prevention practices, antimicrobial stewardship interventions, and shifts in circulating bacterial populations. The decrease observed in early 2024 coincided with a period of intensified infection control efforts; however, causal relationships cannot be established within the retrospective design of this study. The higher proportion of HAIs observed among female patients warrants further investigation and should be interpreted cautiously, as sex-specific differences in exposure or healthcare utilization were not adjusted for.

The separate consideration of ICU-associated cases reflects the distinct clinical and microbiological context of this setting, where patients are exposed to frequent invasive procedures and present a higher burden of comorbidities, factors known to increase the risk of MDR pathogen acquisition. Taken together, the epidemiological and resistance patterns identified in this study reflect broader national and regional trends, including the predominance of MDR Gram-negative pathogens and the concentration of cases in critical care settings. These findings support the need for sustained antimicrobial stewardship, continuous microbiological surveillance, and targeted infection prevention strategies, particularly in high-risk hospital wards such as ICUs.

## 6. Conclusions

This study confirms that MDR-HAIs remain a persistent challenge in a Romanian tertiary care hospital, with a clear concentration of cases in the ICU and a predominance of *A. baumannii* and *Klebsiella* spp. A progressive increase in antimicrobial resistance was observed over time (carbapenems, broad-spectrum cephalosporins, and fluoroquinolones), with resistance rates exceeding 90% during 2023–2024.

In addition to dominant MDR pathogens, the detection of less frequent but highly resistant organisms indicates increasing microbiological complexity, especially among critically ill patients. The sustained ICU-centered distribution of MDR isolates highlights the vulnerability of this setting and the importance of focused surveillance.

Overall, these findings support the need for continuous local resistance monitoring, targeted antimicrobial stewardship, and strengthened infection prevention measures, particularly in high-risk hospital wards.

## Figures and Tables

**Table 1:** Distribution of total HAI samples and MDR-related cases in the study period.

Year	Period	HAI Cases	MDR-Related HAIs	MDR in HAIs (%)
2022	July–December	90	0	0
2023	January–June	93	13	14.0
	July–December	69	25	36.2
2024	January–June	75	18	24.0
Total *		327	56	17.1

* Percentages represent the proportion of MDR-related cases among total HAIs recorded in each corresponding period.

**Table 2:** Age distribution and statistical characteristics.

Age	
Mean	63.9
Median	68
Standard Deviation	18.6
Age range	21–95
Number of patients (*n*)	56

**Table 3:** Patient distribution by hospital ward.

Hospital Ward	Patients (*n*)	Patients (%) *	Median Age (Years)
ICU	33	59	60
Surgical	13	23.2	67
Medical	10	17.9	71
Total	56	100	-

* Percentages represent the proportion of patients within the total study cohort.

**Table 4:** Distribution of MDR-related HAI cases by bacterial strain and hospital ward.

Bacterial Strains	Hospital Wards			
	ICU	Surgical	Medical	Total
*A. baumannii*	6	1	0	7
*Acinetobacter* spp.	6	2	1	9
*Candida lusitaniae*	0 *	1	0	1
*Citrobacter freundii*	0	1	0	1
*E. coli*	0	1	1	2
*Enterobacter aerogenes*	1	0	1	2
*Enterobacter* spp.	0	0	1	1
*K. ozaenae*	4	0	6	10
*K. pneumoniae*	3	1	0	4
*Klebsiella* spp.	1	0	0	1
*Proteus mirabilis*	0	2	0	2
*Providencia stuartii*	2	0	0	2
*P. aeruginosa*	4	0	0	4
*Raoultella* spp.	2	1	0	3
*Serratia marcescens*	3	0	0	3
*S. aureus*	1	3	0	4
Total	33	13	10	56

* Values represent the number of MDR-associated HAI cases. Empty cells indicate zero reported cases.

**Table 5:** Age distribution of patients corresponding to MDR bacterial isolates by semester.

	Age (Year)			
	July–December 2022	January–June 2023	July–December 2023	January–June 2024
Mean	58	67	64	69.2
Median	66	71	66	70
Standard Deviation	20.1	20	15.5	11.9
Jarque–Bera	2.8	2.4	2.7	2.8
Probability	0.2	0.1	0.3	0.3
Count	27	83	131	165

**Table 6:** Median age of patients by semester and area of residence.

Semester	Residence	Median Age (Years)
July–December 2022	Rural	50.6
	Urban	67.2
January–June 2023	Rural	63.7
	Urban	71.8
July–December 2023	Rural	63.3
	Urban	65.2
January–June 2024	Rural	67.6
	Urban	70.7

**Table 7:** Distribution of patients by semester, residence, and gender.

Semester	Residence	Female (*n*)	Male (*n*)	Total (*n*)
July–December 2022	Rural	6	9	15
	Urban	7	5	12
Total	-	13	14	27
January–June 2023	Rural	20	30	50
	Urban	11	22	33
Total	-	31	52	83
July–December 2023	Rural	31	54	85
	Urban	23	23	46
Total	-	54	77	131
January–June 2024	Rural	40	40	80
	Urban	47	38	85
Total	-	87	78	165

**Table 8:** Overview of MDR bacterial genera, associated resistance profiles, and observed temporal patterns (2022–2024).

Bacterial Genus	MDR Isolates (*n*)	Key Resistant Antibiotics (BL, FQ, AMG, C)	Observed Temporal Pattern (2022–2024)
*Klebsiella* spp.	173	AMC, SAM, AMP, FEP, CAZ, CRO, CTX; CIP, LEV; AK; MEM, IPM	Increasing frequency of MDR isolates and broader resistance spectrum from 2023 onward
*Acinetobacter* spp.	83	FEP, CAZ, CRO, CTX, AMC, SAM	Increasing MDR frequency and resistance breadth
*Pseudomonas* spp.	35	FEP, CAZ, CRO; CIP, LEV; IPM, MEM	Relatively stable detection with gradual increase in resistance breadth from 2023 onward
*Enterobacter* spp.	36	AMC, SAM; FEP, CAZ, CRO, CTX; CIP, LEV; IPM	Progressive accumulation of resistance, sporadic C resistance
*E. coli*	11	AMC, SAM; FEP, CAZ, CRO, CTX; CIP, LEV; CN; IPM, MEM	Stable MDR profile across all semesters
*Serratia* spp.	18	FEP, CAZ, CRO, CTX; CIP, LEV, MXF; AK, CN, TOB; IPM, MEM	Progressive increase in resistance breadth and intensity
*Raoultella* spp.	13	AMC, SAM, CAZ, CXM, CRO, CTX, FEP; CIP, LEV; AK, CN; IPM	Predominantly detected in 2023, with clustered occurrence
*Citrobacter* spp.	6	AMC, SAM, AMP; FEP, CAZ, CRO, CTX, CEP; CIP, LEV; AK, CN; ERT, IPM, MEM	Detected from late 2023 with persistence in 2024
*Proteus* spp	16	AMC, SAM, AMP; CAZ, CRO, CTX; CIP, LEV; AK, CN; IPM, MEM; SXT	Persistent detection across all semesters
*Providencia* spp.	11	FEP, CAZ, CRO, CTX; CIP; IPM; SXT; AK, CN	Sporadic detection in 2023 with increase in 2024
*Yersinia* spp.	2	FEP, CAZ, CRO; CIP, LEV; AK, CN; AMC, SAM; IPM	Predominantly detected in July–December 2023, with clustered occurrence
*B. cepacia*	1	FEP, CXM, CRO, CEP; MEM; SXT	Single isolate detected in July–December 2023
*S. maltophilia*	1	LEV; MEM	Single isolate detected in January–June 2024

(BL, β-lactams; FQ, fluoroquinolones; AMG, aminoglycosides; C, carbapenems).

## Data Availability

The original contributions presented in this study are included in the article/Supplementary Material. Further inquiries can be directed to the corresponding author.

## Supplementary Materials

The following supporting information can be downloaded at: https://www.mdpi.com/article/10.3390/jcm15020667/s1, Table S1: Presence of selected patient comorbidities among MDR-associated bacterial isolates (*n* = 56); Table S2: Antibiotic resistance rates by individual antimicrobial agent across the four semesters; Figure S1: Distribution of MDR bacterial isolates by hospital ward (A) and bacterial genus (B) during July–December 2022; Figure S2: Distribution of MDR bacterial isolates by hospital ward (A) and bacterial genus (B) during January–June 2023; Figure S3: Distribution of MDR bacterial isolates by hospital ward (A) and bacterial genus (B) during July–December 2023.

## Abbreviations

The following abbreviations are used in this manuscript:

HAIs	Healthcare-associated infections
MDR	Multidrug-resistant
ICU	Intensive Care Unit
CAUTI	catheter-associated urinary tract infections
CLABSI	central line-associated bloodstream infections
CDI	*Clostridioides difficile* infections
MRSA	methicillin-resistant *Staphylococcus aureus*
CRE	carbapenem-resistant *Enterobacterales*
GCOR	general clinical observation records
SPIAAM	Healthcare-Associated Infection Prevention Service
