# Supplementary material for: Epidemiology of Healthcare-Associated Infections Caused by Multidrug-Resistant Bacteria and Antimicrobial Resistance Patterns in a Romanian Tertiary Care Hospital
Source: J Clin Med. 2026 Jan 14;15(2):667. doi: 10.3390/jcm15020667 (PMC12841648; doi:10.3390/jcm15020667)
Supplement: Supplementary file 1 [file jcm-15-00667-s001.zip › jcm-4029890-supplementary.pdf]

Journal of
Clinical Medicine

MDPI

Table S1 presents the distribution of MDR pathogens according to the presence or absence of selected comorbidities in patients with MDR-HAIs. Arterial hypertension, chronic heart failure, obesity, diabetes mellitus, atrial fibrillation, and oncological conditions were recorded for each patient and analyzed in relation to the isolated bacterial strains.

Oncological conditions were identified in two patients within the cohort. Diabetes mellitus was present in 26.79% of cases. For each comorbidity, the value "1" indicates its presence, while "0" denotes its absence.

Table S1. Distribution of MDR pathogens according to patient comorbidities

**Bacterial strains**	**Diabetes**			**Arterial hypertension**			**Oncological conditions**			**Atrial fibrillation**			**Chronic heart failure**			**Obesity**		
	**0**	**1**	**Total**	**0**	**1**	**Total**	**0**	**1**	**Total**	**0**	**1**	**Total**	**0**	**1**	**Total**	**0**	**1**	**Total**
A. baumannii	7	-	7	6	1	7	7	-	7	6	1	7	6	1	7	7	-	7
Acinetobacter spp.	6	3	9	9	-	9	9	-	9	9	-	9	9	-	9	8	1	9
C. lusitaniae	1	-	1	1	-	1	1	-	1	1	-	1	1	-	1	1	-	1
C. freundii	-	1	1	1	-	1	1	-	1	1	-	1	1	-	1	-	1	1
E. coli	1	1	2	2	-	2	2	-	2	2	-	2	2	-	2	1	1	2
E. aerogenes	2	-	2	2	-	2	1	1	2	2	-	2	2	-	2	2	-	2
Enterobacter spp.	1	-	1	1	-	1	1	-	1	1	-	1	1	-	1	1	-	1
K. ozaenae	8	2	10	7	3	10	10	-	10	9	1	10	10	-	10	9	1	10
K. pneumoniae	4	-	4	3	1	4	4	-	4	3	1	4	3	1	4	3	1	4
Klebsiela spp.	-	1	1	-	1	1	1	-	1	-	1	1	1	-	1	1	-	1
P. mirabilis	1	1	2	1	1	2	2	-	2	1	1	2	2	-	2	2	-	2
P. stuartii	-	2	2	1	1	2	2	-	2	1	1	2	2	-	2	2	-	2
P. aeruginosa	3	1	4	4	-	4	4	-	4	4	-	4	4	-	4	4	-	4
Raoultella spp.	2	1	3	3	-	3	2	1	3	2	1	3	3	-	3	3	-	3
S. marcescens	2	1	3	2	1	3	3	-	3	3	-	3	2	1	3	2	1	3
S. aureus	3	1	4	3	1	4	4	-	4	3	1	4	4	-	4	3	1	4
Total	41	15	56	46	10	56	54	2	56	48	8	56	53	3	56	49	7	56

The value '1' indicates the presence of the specific comorbidity, while '0' denotes its absence.

 https://doi.org/10.3390/jcm15020667

Table S2. Antibiotic resistance rates by individual antimicrobial agent across the four semesters

ANTIBIOTICS	2nd half of 2022		1st half of 2023		2nd half of 2023		1st half of 2024	
	R		R		R		R	
	Nr.	%	Nr.	%	Nr.	%	Nr.	%
**IPM**	11	40,74	71	85,54	122	93,13	153	92,73
**AMC**	21	77,78	70	84,34	98	74,81	104	63,03
**SAM**	19	70,37	64	77,11	113	86,26	154	93,33
**ERT**	6	22,22	58	69,88	85	64,89	104	63,03
**MEM**	21	77,78	78	93,98	120	91,60	153	92,73
**TPZ**	10	37,04	67	80,72	112	85,50	157	95,15
**CZA**	8	29,63	36	43,37	23	17,56	49	29,70
**ATM**	2	7,41	10	12,05	8	6,11	7	4,24
**FEP**	19	70,37	75	90,36	130	99,24	164	99,39
**CAZ**	23	85,19	81	97,59	129	98,47	164	99,39
**CXM**	15	55,56	71	85,54	90	68,70	77	46,67
**CRO**	17	62,96	73	87,95	118	90,08	154	93,33
**CTX**	16	59,26	62	74,70	113	86,26	137	83,03
**CIP**	22	81,48	81	97,59	127	96,95	158	95,76
**LEV**	23	85,19	80	96,39	127	96,95	158	95,76
**NO**	14	51,85	59	71,08	75	57,25	84	50,91
**MXF**	11	40,74	20	24,10	5	3,82	33	20,00
**OFX**	2	7,41	7	8,43	8	6,11	24	14,55
**AK**	21	77,78	70	84,34	99	75,57	140	84,85
**CN**	20	74,07	54	65,06	111	84,73	145	87,88
**TOB**	2	7,41	61	73,49	113	86,26	131	79,39
**SXT**	14	51,85	65	78,31	113	86,26	141	85,45
**C**	2	7,41	41	49,40	78	59,54	92	55,76
**DO**	1	3,70	39	46,99	53	40,46	101	61,21
**TE**	2	7,41	33	39,76	60	45,80	78	47,27
**FOT**	1	3,70	6	7,23	1	0,76	6	3,64
**F**	4	14,81	20	24,10	49	37,40	19	11,52

IPM, imipenem; AMC, amoxicillin–clavulanate; SAM, ampicillin–sulbactam; ERT, ertapenem; MEM, meropenem; TPZ, piperacillin–tazobactam; CZA, ceftazidime–avibactam; ATM, aztreonam; FEP, cefepime; CAZ, ceftazidime; CXM, cefuroxime; CRO, ceftriaxone; CTX, cefotaxime; CIP, ciprofloxacin; LEV, levofloxacin; NOR, norfloxacin; MXF, moxifloxacin; OFX, ofloxacin; AK, amikacin; CN, gentamicin; TOB, tobramycin; SXT, trimethoprim–sulfamethoxazole; C, chloramphenicol; DO, doxycycline; TE, tetracycline; FOT, fosfomycin trometamol; F, nitrofurantoin.

https://doi.org/10.3390/jcm15020667

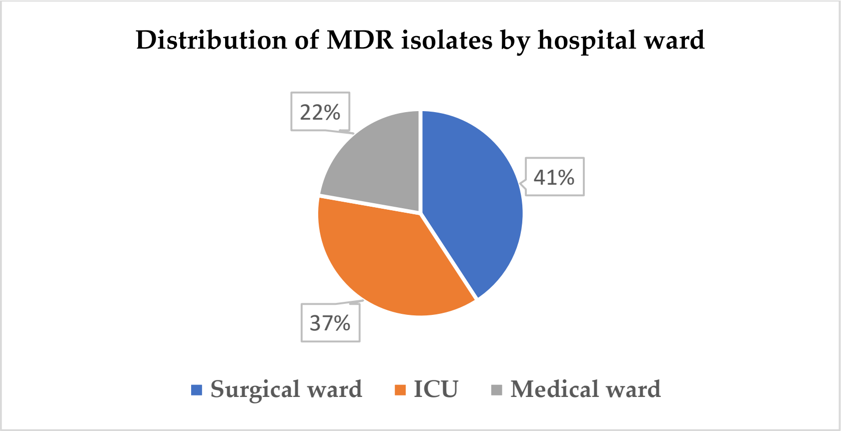

(A)

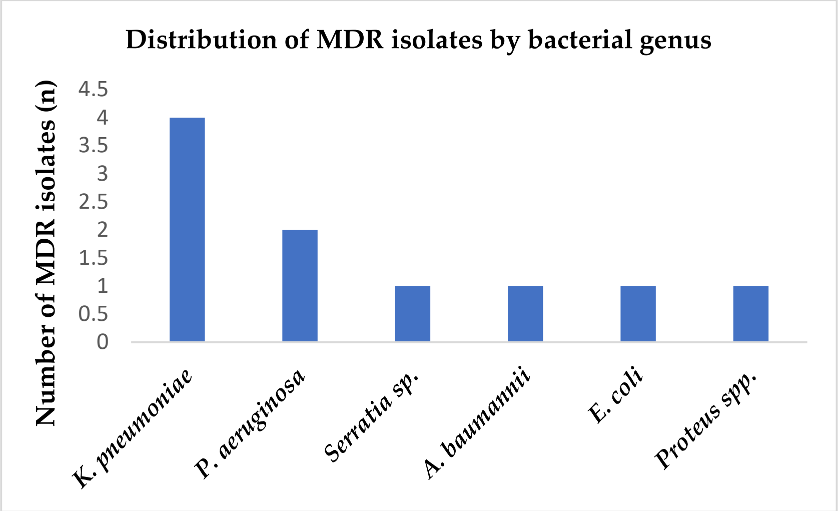

(B)

Figure S1. Distribution of MDR bacterial isolates by hospital ward (A) and bacterial genus (B) during the 2nd half of 2022.

https://doi.org/10.3390/jcm15020667

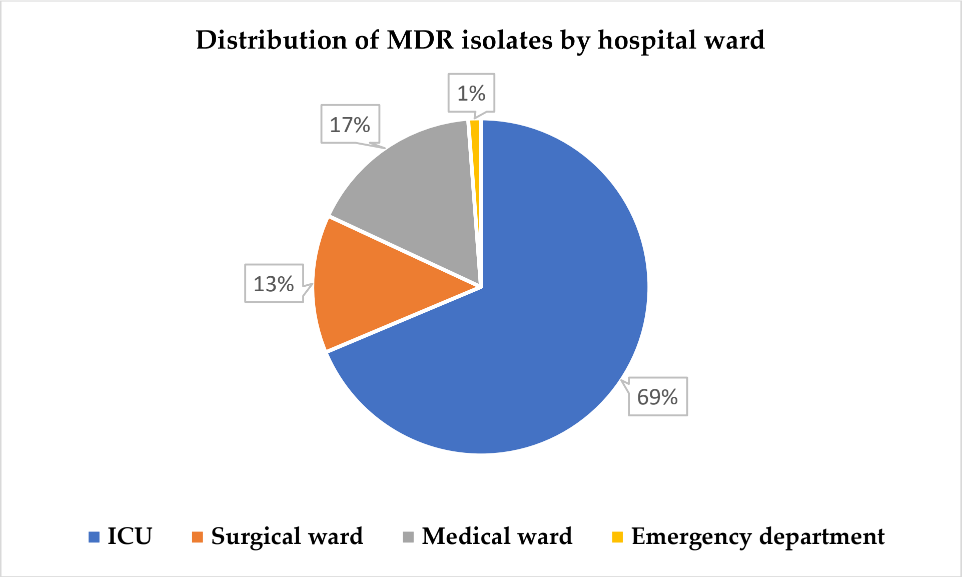

(A)

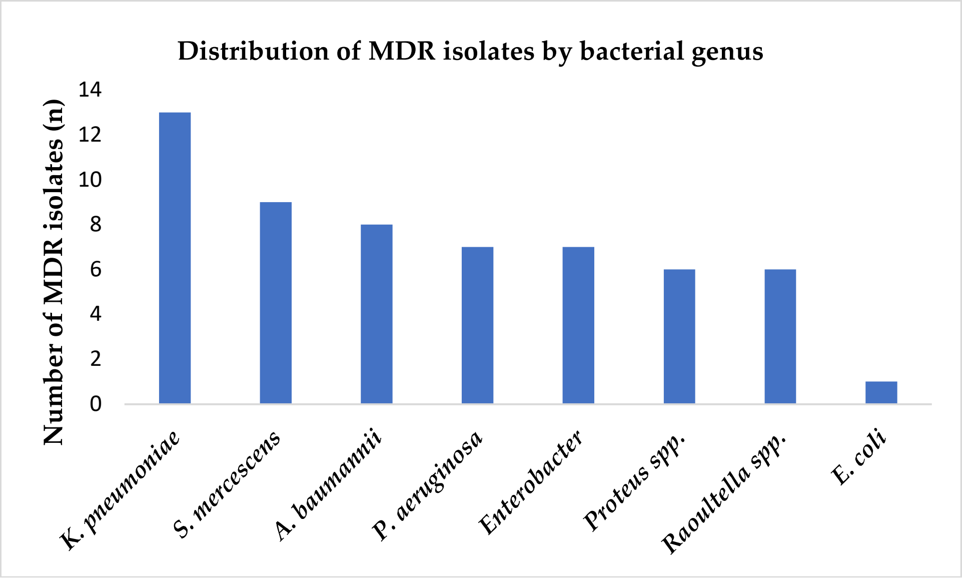

(B)

Figure S2. Distribution of MDR bacterial isolates by hospital ward (A) and bacterial genus (B) during 1st half of 2023

https://doi.org/10.3390/jcm15020667

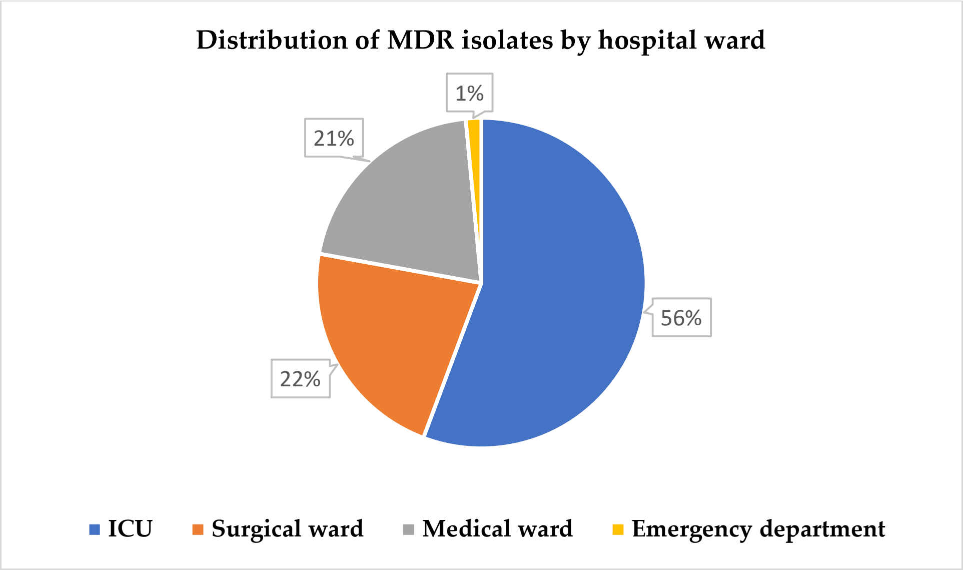

(A)

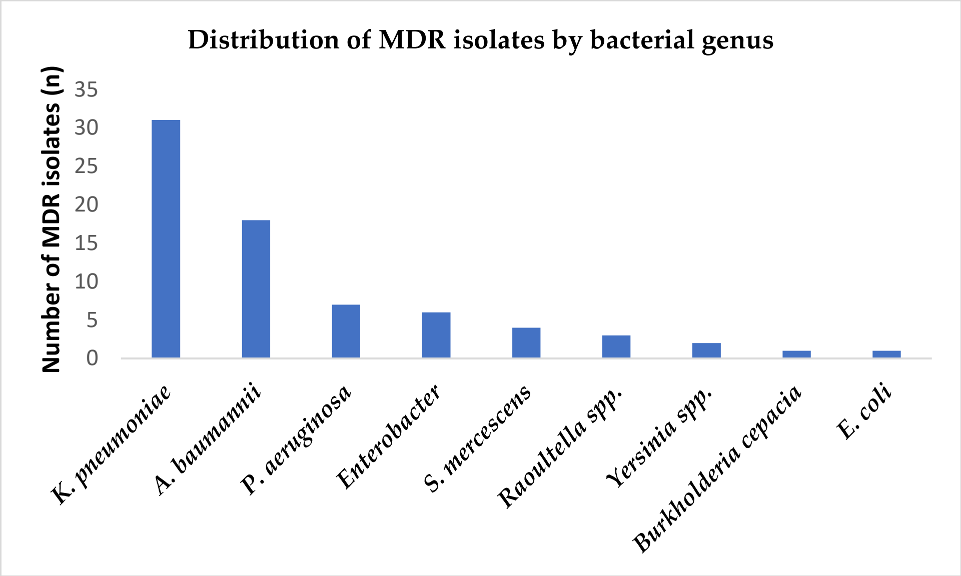

(B)

Figure S3. Distribution of MDR bacterial isolates by hospital ward (A) and bacterial genus (B) during the 2nd half of 2023
